# Supplementary material for: COVID-19-related institutional betrayal associated with trauma symptoms among undergraduate students
Source: PLoS One. 2021 Oct 20;16(10):e0258294. doi: 10.1371/journal.pone.0258294 (PMC8528300; doi:10.1371/journal.pone.0258294)
Supplement: Attachment — Submitted filename: AuthorResponse_PLOSONE_07.11.21.docx [file pone.0258294.s001.docx]

Thank you for taking the time to review our paper entitled “COVID-19-related institutional betrayal associated with trauma symptoms among undergraduate students” for publication in *PLOSONE.* We appreciate the reviewers’ careful consideration of this article and their helpful suggestions and comments. We ensured that the formatting conforms to PLOS ONE’s formatting style. We also included a full ethics statement in the Methods section that denotes the IRB who approved that study, as well as the waiver of written consent. We are excited to submit a revision of this manuscript, incorporating revisions suggested by the reviewers. We believe these revisions have strengthened the manuscript. Our responses to the reviewers’ feedback are contained below.

Sincerely,

Alexis Adams-Clark, M.S.

Jennifer Freyd, Ph.D.

| **Reviewer 1 Comments** | **Author Response** |
| --- | --- |
| Reviewer 1 did not request any revisions | N/A |
| **Reviewer 2 Comments** |  |
| Although the authors provide a definition of institutional betrayal, it might also be helpful to provide a definition of what would count (and not count) as COVID-19 institutional betrayal. Some of the examples provided seemed rather broad…While challenges of remote learning are certainly related to COVID-19, they seem quite different from things the university does that puts students at risk of COVID-19 infection. | Thank you for this comment. This is a great point. Although institutional betrayal can involve a wide range of both actions and inactions, additional information was added on page 4 of the introduction. In addition to greater elucidation of our operationalization of the measurement of institutional betrayal in the Methods section (see below), we hope that this provides some clarification. |
| On page 5, I think "incidence rates of 12 types of COVID-19-related institutional betrayal" should be "prevalence rates of 12 types of COVID-19-related institutional betrayal" as the authors do not have data  on new cases of institutional betrayal. | Thank you for flagging this! We did indeed use the incorrect word. We have changed this to prevalence on page 5. |
| Stronger theoretical justification could be provided for the a priori hypotheses tested in the two studies. For example, why would certain circumstances (e.g., living in a university town, knowing someone infected with COVID-19) be expected to be associated with institutional betrayal ratings? Additionally, although the studies examine intrusion and avoidance cognitions and make specific hypotheses about their associations with institutional betrayal, these cognitions are not defined or discussed in the introduction. | Thank you for this comment. We elaborated on our justifications for these hypotheses in the introduction. (page 5-6) |
| Given that this samples were drawn from a Human Subjects Pool, it would be helpful to also include information about the extent to  which the sample reflects the larger student population of students at  the institution. | Information about the sample representativeness was added to page 7. With the exception of gender (in which women were overrepresented), sample demographics were largely consistent with the university. Although women were over-represented (as is typical in psychology courses and of our Human Subject Pool drawn from such courses), other demographic characteristics of the sample were approximately representative of the university. |
| In initially describing the COVID-19 institutional betrayal measure, it might be helpful to note that the wording of all items appears in Figure 1. When I reviewed the items, some of them seemed specific to COVID-19 (e.g., Created environment where transmission and safety violations seemed common or normal) but others seemed rather broad and could reflect COVID-19 related issues but also more general issues  (e.g., Created environment where continued membership was difficult, Created environment where you no longer felt like a valued member). This speaks to my comments about how COVID-19 institutional betrayal is conceptualized in the study. This could be strengthened so that readers can more clearly understand your operationalization. | Thank you for this comment. Although the text of the items is presented in the figure, several examples were also provided to participants in parentheses. These examples are now described in depth when discussing the IBQ measure in the methods section on pages 10-11, as well as an augmented of institutional betrayal in the introduction section to address the prior comment above. |
| Given that results did not differ without outlier procedures in either study, why was the decision made to cap outliers on the TSC? | This is a good question. We opted to present the results using the capped values in order to provide parameter estimates that are not overly influenced by outliers and more representative of the overall distribution. However, results were run both with and without outliers and statistical conclusions did not differ. |
| In both studies, I was curious why the authors did not control for race/ethnicity in their multiple regression analyses given that systemic racism can lead to disparities in experiences of trauma. | Great question! Race/ethnicity was not associated with significant variance in institutional betrayal, trauma symptoms, or intrusion/avoidance cognitions in either study, so they were not included in the model. We added this clarification to the results section on page 15. In addition, because the majority of our sample is White, there was likely limited power to detect differences, unfortunately. The lack of diversity in the sample is noted in the limitations section. |
| The authors do a nice job presenting potential limitations of their study. It might be useful to also discuss potential issues with common method variance as both COVID-19 institutional betrayal and trauma symptoms are collected via self-report. | This is a great comment. We added a reference to common method variance in our limitations section on page 30 of the manuscript. |
