# Supplementary material for: COVID-19-related institutional betrayal associated with trauma symptoms among undergraduate students
Source: PLoS One. 2021 Oct 20;16(10):e0258294. doi: 10.1371/journal.pone.0258294 (PMC8528300; doi:10.1371/journal.pone.0258294)
Supplement: Attachment — Submitted filename: AuthorResponse_PLOSONE_09.16.21.docx [file pone.0258294.s002.docx]

Thank you for taking the time to review our paper entitled “COVID-19-related institutional betrayal associated with trauma symptoms among undergraduate students” for publication in *PLOSONE.* We appreciate the reviewers’ careful consideration of this article and their receptiveness to our revision. The requested revision by the reviewer was to include demographic statistics for the university community in Table 1. Rather than include it in Table 1, we have decided to instead include additional information in the text of the Methods section regarding specific demographic characteristics of the university student body with citation. This decision was made because publicly available data from the university conflates race and ethnicity, which we separate in our reporting of demographics. They also made several other decisions in the presentation of their data, such as excluding international students, that does not align with our approach. We list these caveats along with the new information we included. In the process of including this information, we also realized that our ethnicity data was missing from Table 1. We included that information in Table 1, so that readers could compare it more readily to the university demographics. We believe these revisions have strengthened the manuscript.

Sincerely,

Alexis Adams-Clark, M.S.

Jennifer Freyd, Ph.D.
